# Dr. Walker, on Dr. Adams's Observations on Cow-Pox

**Published:** 1804-10-01

**Authors:** John Walker

**Affiliations:** Salisbury Square


### *To the Editors of the Medical and Phyſical Journal.*

GENTLEMEN,

WHILE "diseases, like empires, have their revolutions; old ones dying away and ceasing to be heard of, and new ones arising in their place still more formidable and dangerous, as being less understood," the attempts to account for these changes have produced various conjectures. The dreadful Variola has had its rise, its extensive and desolating spread, but now seems happily verging on its extinction. Arising, according to the Arabian authors, in their country, about the time of the commencement of their Hegira, or in the early part of the seventh century of the Christian æra, and first appearing in Egypt in the reign of Omar, the fanatic, who destroyed the library of Alexandria, its origin has been referred to the camel; and after what has been developed in our country, respecting the casual infection of milkers from the cow, the idea may appear plausible. I have often seen the Arabs lying down at night, to take their sleep by the sides of their camels; but is there any other animal which has been longer under domestication than this creature, 'patient of fatigue'? And if from such source, might we not expect some account of the dreadful disease among the patriarchs; some regulations respecting it in the Mosaic institutes; some notice of it by the earliest medical writers of the Greeks? The vulgar notion that Satan inflicted the small-pox on Job, when he smote him with sore boils from the sole of his foot to his crown, must be quite an absurdity. We do not find that any of his friends who came to see him were infected; and there is no mention of their having particularly received a protection from the tormentor.

Perhaps the idea that has been entertained, that the small-pox is no other than degenerated cow-pox, may at last be found to be a correct opinion. I am very naturally led to this surmise by the perusal of the first paper in your last Number. The author, there, (whom I have heard with a great a great deal of pleasure on the subject of cancer, hydatid, &c.) mentions his having inoculated a patient already under a cutaneous affection, three times with vaccine lymph, without producing the local effect; but after she was cured of the first disease, a large number of vesicles appeared in various parts of the body. He says, moreover, "This was not the only instance in which I had reason to believe, that though the local effect of vaccination might be superseded by other causes, yet when those causes ceased, a disposition to the disease, which was formed at the time of insertion, is now brought into action, and shows itself by a general eruption. I very much think that this occurrence is more general than is suspected, and that some of those eruptions which have appeared at a remote period after vaccination, and which the zealots on one side have called small-pox, and those on the other chicken-pox, have been vaccine vesicles. I have already given cases of this arising from a peculiar state of the atmosphere."

Now, if the conjectures be right, that vaccine lymph (vacciolous matter) can under any circumstances produce general eruption; in such departure from its original character we have perhaps one, a first, step of vacciola to its degenerated state of variola. From the earliest pastoral ages, and in various parts of the world, milkers may, in tens of thousands of instances, have received cow-pox; the indisposition produced by it being soon forgotten when it was *once* past. The world at large would, probably, have been ignorant of the existence of such an affection as that of cow-pock in the human subject, had it not been discovered that it protected the system from small-pox. According to the theory of our author, it may have happened in many instances, that the casual inoculation may not have produced its local effect. The milker, already under the influence of some other disease, may have resisted it till such previous disease passed away, when an eruption of vaccine vesicles ought to follow.

If in any constitution, or under any affection of the system, cow-pock could be so modified as to become an eruptive disease, in some disastrous period, we may naturally enough suppose, it may also have become a contagious one; and if so, the most dreadful form of small-pox could not be more horrible than it might immediately become. A case of confluent cow-pox, such pocks as the lancet now produces, were such a thing possible, would as certainly destroy the subject as the act of fleaing him alive.

I presume

I presume the author means by '*after vaccination,*' what I should express by *after insertion of vacciolous matter,* or *after the attempt to vacciolate;* and yet, when he mentions that zealots on one side have called the eruptions after it by one name, and those on the other by another; I am at a loss to comprehend him; because, if it were only *after the attempt,* the advocates of vacciolation would have no cause of surprize or disappointment in finding small-pox; the opponents no triumph. And can the author be correct in referring cases to a peculiar state of the atmosphere? Never having noticed any varieties of appearance or effect produced on vacciola by any diversity of diet, from that of the mother's milk alone to that of salt provisions at sea; nor by any change of atmosphere, from the freshness of the vales of Gloucestershire, or the banks of the Seine, to the dampness of the fields of Holland, or the palpable fogs and smoke of London; from the heat of Gibraltar during a Levanter in Autumn to the coolness of a ship; I cannot help thinking that his manner of accounting for peculiarities must be only hypothetical. Perhaps also his conclusion, that the eruptions in question arose from cow-pock matter, may, after all, have no better foundation than hypothesis.

On the origin of small-pox, which I had hoped I was arriving at through the observations of the author, I find, to use a phrase prevalent in this mercantile country, I am still at sea.

The Author thinks that it would be desirable to have a centre of communication in the metropolis, to which every anomalous case should be referred. The Royal Jennerian Society has such centre: its Medical Council is a Committee, composed of fifty professional men, Physicians, Surgeons, and Apothecaries; perhaps, greater talent than it includes is not to be found in any country. From the beginning it has given notice, that communications of *real importance* would meet with ready attention from it; requesting, that they might be drawn up concisely, and *well authenticated;* and when such communications have been made, they have been respectfully answered, to the satisfaction of the writers. Moreover, the post-masters-general have had the liberality to frank the correspondence of the Society, which amounts, weekly, to several pounds.

Your's, respectfully,

*Salisbury Square,* 16, ix, 1804. JOHN WALKER.

